# A Comprehensive Analysis of Injuries During Army Basic Military Training

**DOI:** 10.1093/milmed/usac184

**Published:** 2022-07-04

**Authors:** Neil Gibson, Jace R Drain, Penelope Larsen, Scott Michael, Herbert Groeller, John A Sampson

**Affiliations:** Centre for Medical and Exercise Physiology, Faculty of Science, Medicine and Health, University of Wollongong, Wollongong, NSW 2522, Australia; Land Division, Defence Science and Technology Group, Fishermans Bend, VIC 3207, Australia; Centre for Medical and Exercise Physiology, Faculty of Science, Medicine and Health, University of Wollongong, Wollongong, NSW 2522, Australia; Centre for Medical and Exercise Physiology, Faculty of Science, Medicine and Health, University of Wollongong, Wollongong, NSW 2522, Australia; Centre for Medical and Exercise Physiology, Faculty of Science, Medicine and Health, University of Wollongong, Wollongong, NSW 2522, Australia; Centre for Medical and Exercise Physiology, Faculty of Science, Medicine and Health, University of Wollongong, Wollongong, NSW 2522, Australia

## Abstract

**Introduction:**

The injury definitions and surveillance methods commonly used in Army basic military training (BMT) research may underestimate the extent of injury. This study therefore aims to obtain a comprehensive understanding of injuries sustained during BMT by employing recording methods to capture all physical complaints.

**Materials and methods:**

Six hundred and forty-six recruits were assessed over the 12-week Australian Army BMT course. Throughout BMT injury, data were recorded via (1) physiotherapy reports following recruit consultation, (2) a member of the research team (third party) present at physical training sessions, and (3) recruit daily self-reports.

**Results:**

Two hundred and thirty-five recruits had ≥1 incident injury recorded by physiotherapists, 365 recruits had ≥1 incident injury recorded by the third party, and 542 recruits reported ≥1 injury-related problems via the self-reported health questionnaire. Six hundred twenty-one, six hundred eighty-seven, and two thousand nine hundred sixty-four incident injuries were recorded from a total of 997 physiotherapy reports, 1,937 third-party reports, and 13,181 self-reported injury-related problems, respectively. The lower extremity was the most commonly injured general body region as indicated by all three recording methods. Overuse accounted for 79% and 76% of documented incident injuries from physiotherapists and the third party, respectively.

**Conclusions:**

This study highlights that injury recording methods impact injury reporting during BMT. The present findings suggest that traditional injury surveillance methods, which rely on medical encounters, underestimate the injury profile during BMT. Considering accurate injury surveillance is fundamental in the sequence of injury prevention, implementing additional injury recording methods during BMT may thus improve injury surveillance and better inform training modifications and injury prevention programs.

## INTRODUCTION

Injury during the initial phase of military training can contribute to attrition and or delayed graduation,^1–3^ impacting upon the supply of personnel to the trained workforce. Globally, the proportion of recruits who sustain at least one injury during basic military training (BMT) varies considerably, with an injury incidence of 20% up to 60% reported.^4–9^ Similarly, within the Australian Army, a wide injury incidence range (14-47%) has been reported during BMT.^10–12^ These global and national discrepancies in injury incidence are likely, at least in part, a result of an inconsistent application of injury definitions^8,9^ and data collection methods.^4,8,10,11^ Nevertheless, musculoskeletal injuries to the lower extremity and lower back are consistently reported as the most common during BMT,^5,7,13,14^ with the majority of injuries diagnosed as overuse (cumulative microtrauma).^7,8,15,16^ However, only recruits who actively seek medical attention (e.g., physiotherapy, nursing, doctor, or medical assistant records)^7,8,15,17^ or experience time-loss (e.g., days of restricted training and hospital days)^1^ are typically captured in injury statistics. Furthermore, overuse injuries can be difficult to capture in epidemiological studies, considering their typical presentation and characteristics.^18^ As such, research to date may underestimate the injury profile during BMT.

In an attempt to improve military injury recording, an injury definition encompassing all injuries is recommended,^19^ yet methods to effectively capture all injuries need to be considered. Herein, self-reported recording methods and external data collectors may facilitate the recording of injuries during BMT. Indeed, self-report methods can allow for the capture of physical complaints whereby individuals may persist with training, without seeking medical attention or experiencing time-loss, despite the presence of injury-associated symptoms and limitations^20^ and have been shown to increase the capture of overuse injuries in athletic populations.^21,22^ Moreover, previous BMT research indicates that injuries most commonly occur during physical training.^16,17,23^ Yet, dedicated physical training sessions only account for 57 hours of a 1,280-hour BMT curriculum (<5%), suggesting other factors likely contribute to the high proportions of overuse injuries reported.^7,8,15,16^ External data collectors (third party) at physical training sessions may therefore assist injury recording by reporting when injuries occur, the suspected mechanism of injury (i.e., overuse or trauma), and by identifying recruits who may not seek medical attention but nevertheless sustain an injury. Although not previously used during BMT, this method has demonstrated utility in the recording of injuries within community sport settings^24,25^ and may provide valuable information with regard to the etiology of injuries reported during physical training as well as the impact of injuries on physical training participation.

The current study therefore aims to capture a more comprehensive understanding of injury incidence during BMT by employing recording methods to capture all physical complaints. This study further aims to evaluate the location and the mechanism of injuries sustained during BMT.

## MATERIALS AND METHODS

### Participants

Recruits from 16 platoons (8 intakes) undertaking BMT at the Army Recruit Training Centre, Kapooka (Australia), during 2019 were invited to participate in the study in week 1 (day 3). Recruits who later joined a platoon within the study (e.g., back squad into a study platoon) were also invited to participate. Before providing written consent, recruits were assured that their participation was voluntary and that participation, or refusal to participate, would have no influence on their training outcome or military career. Six hundred and forty-six recruits (male = 539; female = 107; age: 22 ± 6 years [range:17-55 years]) volunteered to participate (consent rate 95%), 611 recruits (male = 527, female = 84) consented in week 1, 17 recruits (female = 17) from the Army pre-conditioning program consented when joining a platoon in week 3, and 18 “back-squadded” recruits (male = 12, female = 6) consented when joining a study platoon (between weeks 1 and 8). Study procedures were approved by the Department of Defence and Veterans’ Affairs Human Research Ethics Committee (protocol number: 083-18).

### Procedures

A prospective cohort study design was employed. All platoons undertook the standardized 12-week BMT course, involving daily theory and practical lessons, physical training sessions, and a prescribed field training phase. The Australian Army BMT program consists of four key phases, orientation and physical training (weeks 1-3), physical training and military skills (weeks 4-9), field training (weeks 10-11), and ceremonial drill (week 12), with the training demands progressively increasing during the first three phases.^26^ Throughout BMT, injury data were recorded via (1) physiotherapy reports following recruit consultation, (2) a member of the research team (third party) present at physical training sessions, and (3) recruit daily self-reports. On the night of the first self-reported questionnaire, recruits were presented with the injury definition and information relating to questionnaire completion.

#### Injury definition and surveillance

An injury was defined as any physical complaint sustained by a recruit during BMT, irrespective of the need for medical attention or time-loss.^27–29^ Overuse injury was defined as an injury caused by repeated microtrauma without a single identifiable event responsible for the injury, and trauma injury as sudden onset from a specific identifiable event.^28,29^ Physiotherapists at the Army Recruit Training Centre medical centre recorded the date, body part affected, symptoms, injury type, activity when injury occurred (e.g., physical training), mechanism (overuse or trauma), and any prescribed training restrictions for all injuries presented by recruits on each visit using a standardized collection form.

At the end of each day, recruits completed a modified Oslo Sports Trauma Research Centre (OSTRC) Questionnaire on Health Problems,^30^ in a daily diary booklet. The daily diary, consisting of repeated questionnaires for the week (7 days), commenced following week-1 consent (day 3) and continued until the final day of training in week 12 (day 80). The modified OSTRC Questionnaire on Health Problems consisted of five questions: 1 = participation, 2 = severity, 3 = body map to indicate the location of injury (Supplementary Fig. S1, daily modified OSTRC Questionnaire on Health Problems). Questions 2 and 3 were repeated as questions 4 and 5, allowing recruits to indicate their two “worst” health problems daily. Recruits were instructed that injury-related problems should be indicated on the body map, whereas illness-related problems should be documented within the specific check option of “illness” (no location). A “no injury/illness” check option was also provided to ensure that an answer was required for all questions.

A member of the research team (third party), who held a minimum qualification level of Bachelor of Science in a Sport and Exercise Science–related discipline, was present at all physical training sessions (40 sessions per platoon, total = 320 sessions) to record all self-reported recruit complaints. At the beginning of each physical training session, recruits with training restrictions and recruit absences were noted. In the event of an injury, and for recruits presenting at the start of physical training as injured or unwell, the third party completed the same standardized data collection form used by physiotherapists. Any prescribed restrictions, recruit training modifications or time missed (e.g., recruit arrived late), were also recorded. At the completion of each physical training session, recruits were asked if any injuries were sustained during the session and the standardized collection form completed if not already captured. If a recruit presented with numerous injuries, all injuries were recorded. All musculoskeletal injuries were recorded as “non-specific pain.”

### Data Preparation and Analyses

Data collection forms (from physiotherapists and third party) were routinely collected and manually processed throughout the study. Daily self-reports were scanned (Canon DRC240, Canon, Tokyo, Japan) and subsequently processed using Remark Office OMR software (Remark, Malvern, PA, USA). All data were entered and saved in password-protected Excel workbooks.

Third-party and self-reports identifying only “illness” were excluded from analyses. Any self-report with a body map location checked was classified as an injury-related report. To calculate compliance and the total number of training days, self-reports were matched to all potential days in training. Recruits who left a platoon and joined another platoon in the study (e.g., back-squadded) were accounted for (i.e., additional training days). If a recruit joined the study late or left the study (e.g., pending discharge), only days in a study training platoon were included in the analyses.

Physiotherapist and third-party reports were linked to an International Classification of Disease, tenth revision, Clinical Modification (ICD-10 CM) code (to six digits when available, minimum four digits), based on the body part affected and type of injury. An injury was defined as the first or incident occurrence of a specific injury (i.e., ICD-10 CM code). To avoid overestimating injuries, at least 30 days had to pass after an individual’s last report with a specific ICD-10 CM code (30-day “gap” rule), before that code could again be counted as a new “incident injury.”^7,19^ Self-reported injury locations were aligned with body locations from the taxonomy,^19^ and the 30-day “gap” rule was applied to specific body locations (e.g., right arm, upper). Incident injury frequencies categorized by the general (e.g., lower extremity) and specific body region (e.g., arm, upper) were calculated for each recording method.^19^ Injury mechanism and type, as indicated by physiotherapist and third party, were also analyzed. Incident injuries that occurred and were recorded by the third party during the same physical training session were analyzed to assess physical training session injuries.

Injury incidence (%) was calculated as per equation (1)^8,19^:


(1) $$&Injury\ incidence\ \left( \% \right) \nonumber\\&= {{{\mathrm{number\ of\ recruits\ with\ one\ or\ more\ incident\ injuries}}} \over {{\mathrm{total\ number\ of\ recruits}}}} \nonumber\\& \quad \times 100$$

To account for recruit attrition over the course of BMT and the potential for recruits to repeat training days (e.g., back-squadding), days in training were also considered. Person-time injury incidence, expressed as recruits injured per 100 person-days, was calculated as per equation (2)^15,31^:


(2) $$&Person\ time\ injury\ incidence \nonumber\\&= {{{\mathrm{number\ of\ recruits\ with\ one\ or\ more\ incident\ injuries}}} \over {{\mathrm{total\ number\ of\ days\ in\ a\ training\ platoon\ within\ the\ study}}}} \nonumber\\& \quad \times 100$$

To investigate the distribution of incident injuries, while accounting for recruit attrition and new recruits joining, injury incidence per week per 100 recruits was calculated as per equation (3):


(3) $$&Incident\ injuries\ per\ week\ per\ 100\ recruits\ \nonumber\\&= {{{\mathrm{number\ of\ incident\ injuries\ in\ week\ of\ training}}} \over {{\mathrm{number\ of\ recruits\ in\ week\ of\ training}}}} \times 100$$

Physical training time-loss, defined as an inability to fully participate in physical training, was categorized as (1) partial time-loss (participated, but missed a specific training component, or performed modified training) and (2) total time-loss (did not actively participate). When recruits were not present at physical training, time-loss was recorded with the reported reason “not confirmed.” For self-reports, an accumulated injury score was not calculated,^30^ as a modified OSTRC Questionnaire on Health Problems was used, rather the “participation” (question 1) and “severity” (questions 2 and 4) categories were analyzed.^32^ All data preparation and analyses were performed with *R* (version 4.0.2, R Foundation for Statistical Computing, Vienna, Austria).

## RESULTS

For the 646 recruits, there were a total of 46,197 (male = 38,937, female = 7,260) individual recruit training days with an average of 72 days (min = 4, Q1 = 80, median = 80, Q3 = 80, max = 101 days) in training per recruit. The response rate to the daily health questionnaire was 90.5%. In all, 235 recruits had ≥1 incident injury recorded by physiotherapists, 365 recruits had ≥1 incident injury recorded by the third party, and 542 recruits reported ≥1 injury-related problems via the self-reported health questionnaire. Subsequently, an injury incidence of 36.4%, 56.5%, and 83.9% and person-time injury incidence of 0.51, 0.79, and 1.17 recruits injured per 100 person-days were observed for physiotherapy, third party, and self-reports, respectively.

**FIGURE 1.**
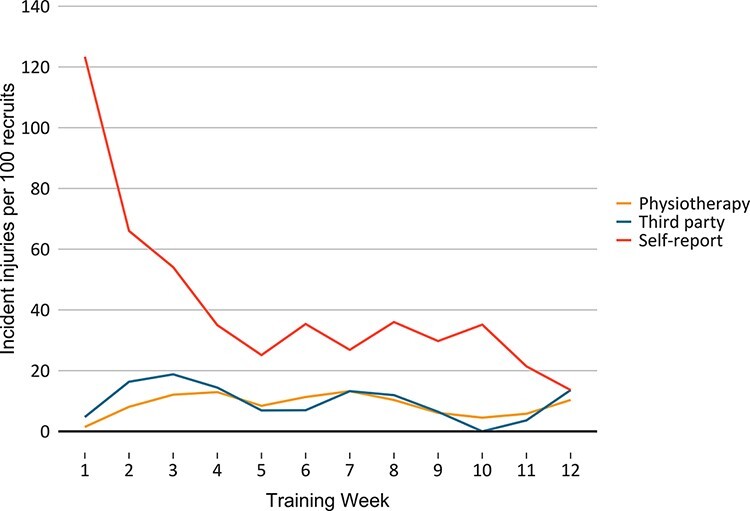
Incident injuries per week of basic military training, expressed relative to the number of recruits in the specific week of training and multiplied by 100 to indicate incident injuries per week per 100 recruits.

In all, 621, 687, and 2,964 incident injuries were recorded from a total of 997 physiotherapy reports, 1,937 third-party reports, and 13,181 self-reported injury-related problems, respectively. Incident injuries per week of BMT are illustrated in Figure 1. Overuse accounted for 79% and 76% of documented injuries from physiotherapist (493/621) and third party (525/687), respectively. All three recording methods indicate that the lower extremity was the most commonly injured general body region (Table I). Incident injury types provided by physiotherapists and the third party are summarized in Tables II and III (Supplementary Table S1, ICD-10 CM codes).

**TABLE I.** Incident Injuries as Captured by Each of the Three (Physiotherapy, Third Party, and Self-Reports) Injury Recording Methods

Incident injuries												
	Physiotherapy					Third party					Self-report	
			Overuse or trauma					Overuse or trauma				
	Total	Total (%)	Overuse	Trauma	Unspecified	Total	Total (%)	Overuse	Trauma	Unspecified	Total	Total (%)
Head and neck												
Other head	0	0.0	0	0	0	9	1.3	0	9	0	134	4.5
Face	0	0.0	0	0	0	1	0.2	0	1	0	–	–
Neck	1	0.2	1	0	0	0	0.0	0	0	0	78	2.6
Total for the body region	1	0.2	1	0	0	10	1.5	0	10	0	212	7.2
Spine and back												
Back, upper	1	0.2	1	0	0	15	2.2	14	1	0	–	–
Back, middle	9	1.5	7	0	2	14	2.0	11	2	1	83	2.8
Back, lower	43	6.9	31	11	1	50	7.3	39	10	1	164	5.5
Total for the body region	53	8.5	39	11	3	79	11.5	64	13	2	247	8.3
Torso												
Chest	12	1.9	7	5	0	11	1.6	7	3	1	34	1.2
Abdomen	1	0.2	1	0	0	1	0.2	1	0	0	42	1.4
Pelvis	4	0.6	3	1	0	7	1.0	6	1	0	–	–
Trunk, other	0	0.0	0	0	0	0	0.0	0	0	0	57	1.9
Total for the body region	17	2.7	11	6	0	19	2.8	14	4	1	133	4.5
Upper extremity												
Shoulder	47	7.6	35	10	2	36	5.2	31	5	0	228	7.7
Arm, upper	2	0.3	2	0	0	11	1.6	6	5	0	297	10.0
Elbow	3	0.5	2	1	0	17	2.5	12	5	0	96	3.2
Arm, lower	16	2.6	9	5	2	4	0.6	1	3	0	27	0.9
Wrist	1	0.2	0	1	0	7	1.0	5	2	0	83	2.8
Hand, finger	0	0.0	0	0	0	17	2.5	5	11	1		
Total for the body region	69	11.1	48	17	4	92	13.4	60	31	1	731	24.7
Lower extremity												
Hip	71	11.4	65	3	3	50	7.3	42	8	0	59	2.0
Leg, upper	20	3.2	15	5	0	35	5.1	27	6	2	305	10.3
Knee	87	14.0	70	17	0	113	16.5	79	30	4	413	13.9
Leg, lower	176	28.3	154	13	9	110	16.0	103	5	2	353	11.9
Ankle	39	6.3	14	25	0	57	8.3	27	29	1	511	17.2
Foot, toe	86	13.9	75	4	7	122	17.8	109	11	2		
Total for the body region	479	77.1	393	67	19	487	70.9	387	89	11	1641	55.4
Other												
Unspecified	2	0.3	1	1	0	0	0.0	0	0	0	–	–
Total for the body region	2	0.3	1	1	0	0	0.0	0	0	0	–	–
All injuries	621^a^	100	493	102	26	687^b^	100	525	147	15	2,964^c^	100

“-” indicates specific body locations that were not included within the self-reports. The underline value indicates where two body locations were combined within the self-report injury questionnaire.

a Initial encounters (up to the sixth digit, 30-day gap rule) calculated from a total of 999 injury reports.

b Initial encounters (up to the sixth digit, 30-day gap rule) calculated from a total of 1,937 injury reports.

c Initial encounters (based on taxonomy-specific injury locations, 30-day gap rule) calculated from a total of 13,182 self-reported complaints. Specific body locations with no injuries are not shown.

**TABLE II.** Incident Injury Type as Reported by Physiotherapists

Physiotherapy injury data		
General body region	Type of injury	Total
Head and neck	Muscle rupture/strain/tear	1
Spine and back	Muscle rupture/strain/tear	19
	Non-specific pain	32
	Unspecified	2
Torso	Stress fracture/reaction	3
	Other bone injuries	2
	Muscle rupture/strain/tear	7
	Hematoma/contusion/bruising	1
	Non-specific pain	2
	Other	1
	Unspecified	1
Upper extremity	Dislocation/subluxation	3
	Muscle rupture/strain/tear	26
	Sprain/ligament	2
	Tendon rupture/tendinosis/bursitis	6
	Hematoma/contusion/bruising	1
	Non-specific pain	18
	Other	11
	Unspecified	2
Lower extremity	Fracture	1
	Stress fracture/reaction	31
	Dislocation/subluxation	5
	Other bone injuries	5
	Muscle rupture/strain/tear	126
	Sprain/ligament	28
	Tendon rupture/tendinosis/bursitis	39
	Hematoma/contusion/bruising	14
	Abrasion/graze/blister	8
	Cramp	2
	Non-specific pain	175
	Other	39
	Unspecified	6
Other	Non-specific pain	1
	Other	1

**TABLE III.** Incident Injury Type as Reported by Third Party, Including Physical Training Time-Loss (Partial and Total) for General Body Region and Type of Injury

Third-Party Injury Data				
			Physical training time-loss	
General body region	Type of injury	Incident injuries	Partial time-loss^a^	Total time-loss
Head and neck	Non-specific pain	2	0	0
	Abrasion/graze/blister	2	0	0
	Hematoma/contusion/bruising	1	0	0
	Laceration/cut	4	0	0
	Other—tooth	1	0	0
Spine and back	Non-specific pain	78	12	15
	Unspecified	1	0	0
Torso	Non-specific pain	17	1	3
	Hematoma/contusion/bruising	2	2	2
Upper extremity	Non-specific pain	78	25	5
	Abrasion/graze/blister	1	0	0
	Laceration/cut	8	0	1
	Burn	5	0	0
Lower extremity	Non-specific pain	394	85	60
	Abrasion/graze/blister	35	5	1
	Hematoma/contusion/bruising	20	4	2
	Laceration/cut	4	0	0
	Other—ingrown toenail	18	5	3
	Other—foot rash	14	1	2
	Unspecified	2	1	0

a On occasions, partial time-loss in physical training was attributable to more than one injury. Therefore, although 136 instances of partial time-loss were reported, partial time-loss in this table totals 141, as if two injuries were recorded both are reported.

There were 1,166 third-party reports of a recruit on training restrictions at physical training, from a total of 263 different recruits who presented with ≥1 injury-related training restriction. Of the 1,211 instances of time-loss at physical training, the reason was “not confirmed” on 918 occasions (76%). On 293 occasions (24%), the reason for time-loss was known, with injury being the cause on 230 occasions, consisting of 136 instances of partial and 94 instances of total time-loss (Table III). In total, 147 recruits had known time-loss at physical training because of injury. Of the 687 incident injuries recorded by the third party, 252 occurred and were subsequently recorded during the physical training session being observed. Of these, 184 (73%) were recorded as overuse (Supplementary Table S2, physical training injuries).

On days when recruits self-reported a problem (8,894 days), there were 7,042 self-reports of “full participation but with an injury,” 631 self-reports of “reduced participation due to injury”, and 157 self-reports of “could not participate due to injury.” Despite indicating a problem on the body map, “full participation without injury” was reported on 1,050 occasions, while on 14 occasions, no response to this question was provided. Of the 13,181 self-reported problems recorded, 7,615 were recorded as “mild,” 3,128 as “moderate,” 586 as “severe,” and on 233 occasions recruits indicated that they “could not participate.” On 232 occasions, no response to the severity question (question 2 or 4) was provided despite a reported problem on the body map, and 1,382 problems with a body location checked were reported as “no symptoms/health-problems.” On five occasions, recruits indicated that they “could not participate” although this was not indicated in Q1.

## DISCUSSION

This study highlights that injury surveillance methods impact injury recording during BMT with injury incidence from the third party and recruit self-reports 1.5 and 2.3 times greater than physiotherapy reports, respectively. Traditional injury surveillance methods, which rely on medical encounters, thus fail to capture the magnitude of injuries sustained during BMT. Nevertheless, the lower extremity was consistently recorded as the most commonly injured body region across all three recording methods, while physiotherapy and third-party reports indicate that the majority of incident injuries were overuse related. Self-reports recorded the greatest number of incident injuries and although injury type and mechanism could not be determined, this finding likely indicates that a substantial number of overuse problems are not captured by traditional injury recording methods. Furthermore, third-party reports highlight that 73% of injuries that were sustained and recorded within the same physical training session were overuse related. Thus, although injury-related symptoms may arise or develop during physical training, the cause is likely to be multifarious and should not be solely attributable to physical training.

It was anticipated that physiotherapy reports would not capture all injuries during BMT, as this surveillance method requires recruits to seek medical attention.^33,34^ Physiotherapy reported injury incidence (36.4%) and incident injury locations (lower extremities, 77.1%; shoulder, 7.6%; and lower back, 6.9%) were nevertheless relatively central to the range, and consistent with injury locations reported during BMT internationally.^6–8,14,16^ Indeed, injury data retrieved for Australian Army recruits over a 5-year period (2006-2011) reported a similar injury incidence (34.3%), despite differences in data collection methods (e.g., physiotherapists, medical staff, and physical training instructors).^11^ The most common injury mechanism captured in physiotherapy reports during this study was overuse (79%), a finding that is consistent across the literature,^3,7,16^ while the specific injury type of “non-specific pain” (37%) and “muscle rupture/strain/tear” (29%) also aligns with research highlighting “pain” as the most common injury diagnosis in recruit populations^35^ and the prevalence of soft-tissue injuries during BMT.^16^

Previous research has however indicated that recruits may not always seek medical attention for an injury, whereas others may delay reporting, in an attempt to defer medical attention or time-loss, in order to graduate from BMT on time.^33^ It was also considered that overuse injuries, which typically present with symptoms such as pain or functional limitation and often appear gradually and may even be transient in nature,^21,27^ would likely be underreported during BMT as recruits may continue to train despite the presence of overuse problems. Indeed, a far greater number of incident injuries were recorded using the modified OSTRC Questionnaire on Health Problems within the present study (four times as many), capturing more recruits within injury statistics. This finding may appear to contradict previous research, where self-reports were shown to underestimate injury incidence in U.S. Army Infantry soldiers when compared with outpatient medical records.^36^ However, the 12-month recall period likely affected the outcomes reported by Schuh-Renner and colleagues.^37^ In contrast, self-reports were completed daily in the current investigation in an attempt to improve accuracy.^20^ Furthermore, the daily diary was not viewed by training staff, and responses had no influence upon recruits’ training progression. Separating injury reports from the evaluation of military performance likely contributed to the increased reporting observed within the present study^34^; however, underreporting cannot be discounted as recruits may have been reluctant to report if they perceived staff may use reports to monitor their response to BMT.^33^

Self-reports also highlight the lower extremity as the most commonly injured general body region. The relative percentage of lower extremity incident injuries was however lower than physiotherapy reports (55.4% vs. 77.1%, respectively). This discrepancy may be attributable to the increased capture of overuse injuries in daily self-reports,^21,22,30^ but as only the anatomical location of problems was reported this cannot be confirmed. Considering the broad injury definition used, trauma-related injuries (e.g., acute) are also likely to have been captured by self-reports.^30^ Follow-up consultations with recruits to classify the type and mechanism of self-reported problems could address this limitation.^21,30^ Yet, it should also be acknowledged that the majority of self-reported problems captured within the present study were mild (57.7%) or moderate (23.7%), and as such, likely encapsulated less severe injuries, which may not have warranted medical attention. Furthermore, as self-reports reflect recruits’ self-assessment (e.g., perception) of pain, “normal” (e.g., delayed onset-muscle soreness) training-related pain may also be captured in these reports.^21^ The value that can be gained from this additional information should however not be underestimated, as the reporting of a problem likely reflects the presence of pain,^32^ which can gradually develop into a more serious injury.^35,38^

The greatest number of self-reported incident injuries were observed in week 1, likely a response to the new, potentially unfamiliar, stressors of BMT.^3,13^ It is also important to recognize that any reported problem would likely have been the first (incident occurrence) to that specific body location. Appropriately, this may explain the steep decline in incident injuries following week 1, given the 30-day gap rule was used to avoid overestimating incident injuries.^19,35^ Consequently, a new incident injury to the same specific body location within this period may have been missed. Notably, a far greater number of incident injuries to the upper arm were recorded by self-reports, in comparison to physiotherapy reports, although 212 of the 297 incident injuries (71%) were recorded in week 1, when recruits received inoculations. Previous research has suggested that the first week of reporting can result in an artificially high number of cases,^21^ while familiarization with the questionnaire and injury definition may have also impacted reporting within the present study. Indeed, on numerous occasions, recruits checked a body location indicative of an injury but did not indicate this in the preceding questions (i.e., participation or severity), potentially because of recruits not perceiving their problem to be an injury but reporting the location of injury-related symptoms (e.g., pain).

Consistent with the documented underreporting of injuries in a military environment,^34,39^ third-party reports recorded a greater injury incidence and number of incident injuries in comparison to physiotherapy reports. Incident injuries per general body region documented by the third party were, in general, similarly distributed when compared with physiotherapy reports; however, the third party captured a greater number of injuries to the head and neck region. All third party recorded head and neck injuries were trauma-related (e.g., laceration); therefore, these discrepancies are likely, at least in part, attributable to the third party capturing a greater variety of injuries (e.g., lacerations) that may not have required consultation with a physiotherapist. It is also possible that the presence of the third party at physical training sessions offered a more accessible means of reporting. Yet, recruits still had to report to the third party while in the presence of physical training instructors, recruit instructors, and their peers; thus, perceptions or stigmas associated with injury likely remained across both recording methods.^6,9,33,40^ Unless superficial, all third party documented injuries were diagnosed as “non-specific pain,” which subsequently accounted for 83% of incident injuries. Despite the lack of a clear diagnosis, 76% of the third party reported incident injuries were classified as overuse, which was strikingly similar to the physiotherapist reports (79%). More specifically, 73% of injuries observed during physical training were overuse related. Thus, while activities performed in physical training (e.g., running) may aggravate existing symptoms (e.g., pain),^23^ the underlying cause is likely a consequence of the collective stress of military training. In fact, in overuse injuries, tissue failure can already be present before the development of pain or performance decrements.^41^

Weekly incident injuries recorded by the third party and physiotherapy methods tracked relatively similarly throughout BMT, potentially because of the third-party recording recruits who were on training restrictions, which were likely provided by the physiotherapists. In weeks when fewer incident injuries were recorded by the third party in comparison to physiotherapy, limited physical training sessions (≤3 sessions per week) were conducted; accordingly, as no physical training sessions were scheduled in week 10, no third-party incident injuries were recorded. Finally, a proposed benefit of the third party was to assess the impact of injury on physical training participation; however, on numerous occasions (*n *= 918), recruits were not present at physical training; therefore, the reason for time-loss (e.g., injury, illness, and appointments) could not be confirmed. As such the impact of injury on physical training participation could not be determined.

### Limitations

When interpreting these results, several limitations should be acknowledged. First, as it was not feasible to implement at all point-of-care facilities, only physiotherapists completed standardized collection forms for each recruit consultation and as such medical-attention incident injuries may have been underestimated. However, comparisons between previous and current results suggest that physiotherapists capture the majority of recruits who seek medical attention.^11^ Furthermore, in an attempt to standardize injury reporting, physiotherapy and third-party reports were aligned with ICD-10 CM codes, before applying a 30-day “gap” rule to calculate incident injuries.^7,19^ The effect that this rule may have had on the self-reported injuries is discussed. However, this rule also likely impacted physiotherapy reports as information relating to initial and subsequent encounters were not obtained.^19^ Consequently, physiotherapy variance when diagnosing an injury may have impacted upon the number of incident injuries calculated,^19,40^ potentially overestimating incident injuries. In contrast, the third party consistently reported all non-superficial injuries as “non-specific pain,” thus possibly underestimating incident injuries. Inaccuracies in self-reported data were also possible, with recruits frequently checking a location on the body map for injury-related problems, while also checking “illness” (*n *= 2,464 reports). These injury locations were included in the injury analyses and may have resulted in an over-reporting of incident injuries. Additionally, although questionnaires were designed to be completed daily, recruits may have recalled information from previous days before the weekly diary collection.

## CONCLUSION

The present findings suggest that traditional injury surveillance methods underestimate the injury profile during BMT; however, consistent with previous reports these data confirm the lower extremity as the most injured body region and overuse as the most common injury mechanism. Considering accurate injury surveillance is fundamental in the sequence of effective injury prevention,^42^ an inability to record all injuries can prevent progress toward prevention. As such, the implementation of additional injury surveillance methods during BMT may improve injury reporting and help inform training modifications and injury prevention programs. Although the costs associated with implementing additional injury surveillance should be recognized, it is also important to acknowledge the costs associated with enlisting and training recruits, irrespective of whether they complete training or not.^2,43^ Considering injury during BMT can result in lengthy periods of rehabilitation, delayed training completion, and discharge,^13,44^ there is arguably a compelling economic argument for improved injury surveillance to help minimize training disruption and reduce the costs associated with injury and attrition.^2,43^ Although typically used as an injury surveillance tool, the OSTRC questionnaires have also been suggested as a potential daily monitoring tool,^20^ and in sport the risk of sustaining a “time-loss” injury appears amplified if preceded by a self-reported injury-related problem.^32^ Further examination of the use of self-reported injury data as a systematic recruit monitoring strategy may therefore be beneficial.

## Supplementary Material

usac184_Supp

## Data Availability

The datasets generated and analyzed during the current study are not publicly available. Data will only be made available upon formal request to the corresponding author who will seek approval from the relevant agencies.
