# Supplementary material for: A Comprehensive Analysis of Injuries During Army Basic Military Training
Source: Mil Med. 2022 Jul 4;189(3-4):652–60. doi: 10.1093/milmed/usac184 (PMC10898870; doi:10.1093/milmed/usac184)
Supplement: usac184_Supp [file usac184_supp.zip › Supplementary Figure 1.pdf]

WEEK 1 – Day 3 - Thursday

Daily Self-Reported Injury Questionnaire (EVENING)

INSTRUCTION: Please fill the entire circle to mark your answer. e.g. ●

If you have several injury or illness problems please refer to the one that has been your worst problem today. You will have a chance to register an additional problem on the next page (Q4-5).

Question 1 - Have you had any difficulties partaking in military training / activities / education due to injury, illness or other health problems today?

- ○ Full participation without injury/illness
- ○ Full participation, but with injury/illness
- ○ Reduced participation due to injury/illness
- ○ Could not participate due to injury/illness

Question 2 - To what extent have you experienced symptoms / health complaints today?

- ○ No symptoms/health problems
- ○ To a mild extent
- ○ To a moderate extent
- ○ To a severe extent
- ○ Could not participate at all

Question 3 - On the image below please select **one** circle that best describes the location of your **injury**. ****If your problem is related to an illness please fill the illness circle. If you do not have an injury / illness please fill the no injury / illness circle*****

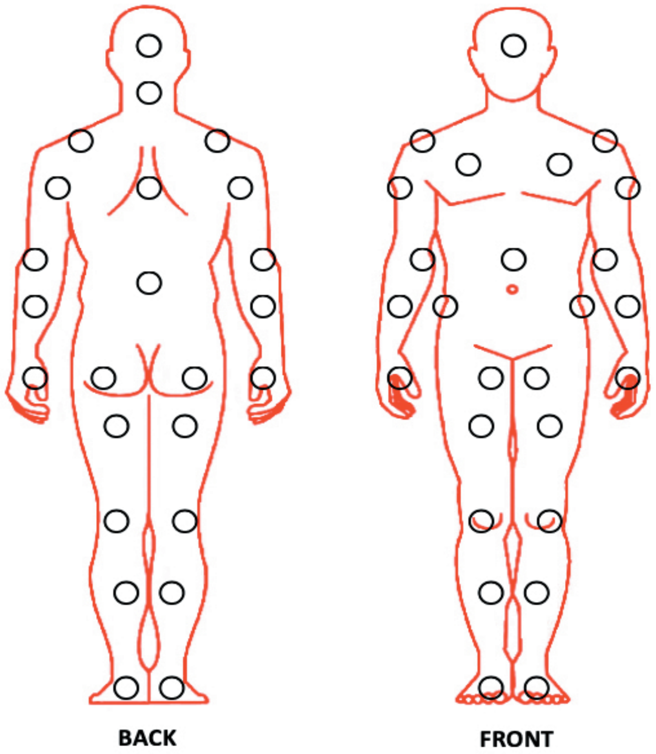

- ○ Illness
- ○ No injury / illness
