# Supplementary material for: A Comprehensive Analysis of Injuries During Army Basic Military Training
Source: Mil Med. 2022 Jul 4;189(3-4):652–60. doi: 10.1093/milmed/usac184 (PMC10898870; doi:10.1093/milmed/usac184)
Supplement: usac184_Supp [file usac184_supp.zip › Supplementary Table 1.pdf]

ICD-10-CM Code		Description	Total
M2556 (1, 2, 9)		Pain in knee (right, left, unspecified)	66
M7967 (1, 2, 3)		Pain in foot (right, left, unspecified)	42
S7601 (1, 2, 9)		Strain of muscle, fascia and tendon of hip (right, left, unspecified)	37
M7966 (1, 2, 9)		Pain in lower leg (right, left, unspecified)	32
S8691 (1, 2, 9)		Strain of unspecified muscle(s) and tendon(s) at lower leg level (right, left, unspecified)	31
S9340 (1, 2, 9)		Sprain of unspecified ligament of ankle	23
M545		Low back pain	22
M2555 (1, 2, 9)		Pain in hip (right, left, unspecified)	21
S9691 (1, 2, 9)		Strain of unspecified muscle and tendon at ankle and foot level (right, left, unspecified)	21
M84369		Stress fracture, unspecified tibia and fibula	20
S39012		Strain of muscle, fascia and tendon of lower back	19
S4691 (1, 2, 9)		Strain of unspecified muscle, fascia and tendon at shoulder and upper arm level (right, left, unspecified)	19
M2551 (1, 2, 9)		Pain in shoulder (right, left, unspecified)	16
S9691 (1, 2, 9) / M779 / M719	#	Strain of unspecified muscle and tendon at ankle and foot level (right, left, unspecified) / Unspecified enthesopathy, lower limb, excluding foot / Bursopathy, unspecified	15
M2557 (1, 2, 9)		Pain in ankle and joints of foot (right, left, unspecified)	13
S899 (0, 1, 2)		Unspecified injury of lower leg (unspecified, right, left)	12
S8621 (1, 2, 9)	#	Strain of muscle(s) and tendon(s) of anterior muscle group at lower leg level (right, left, unspecified)	11
S8691 (1, 2, 9) / M769 / M705 (0, 1, 2)		Strain of unspecified muscle(s) and tendon(s) at lower leg level (right, left, unspecified) / Unspecified enthesopathy, lower limb, excluding foot / Other bursitis of knee (unspecified, right, left)	10
S9992 (1, 2, 9)		Unspecified injury of foot (right, left, unspecified)	10
M546		Pain in thoracic spine	9
S7611 (1, 2, 9)		Strain of quadriceps muscle, fascia and tendon (right, left, unspecified)	9
S7631 (1, 2, 9)		Strain of muscle, fascia and tendon of the posterior muscle group at thigh level (right, left, unspecified)	9
M8437 (4, 5, 6)		Stress fracture, foot (right, left, unspecified)	8
S8611 (1, 2, 9)		Strain of other muscle(s) and tendon(s) of posterior muscle group at lower leg level (right, left, unspecified)	8
S9081 (1, 2, 9) / S9082 (1, 2, 9)	#	Abrasion foot (right, left, unspecified) / blister (nonthermal) foot (right, left, unspecified)	8
M222X (1, 2, 9)		Patellofemoral disorders, knee (right, left, unspecified)	7
S800 (0, 1, 2)		Contusion of knee (unspecified, right, left)	7
S903 (0, 1, 2)		Contusion of foot (unspecified, right, left)	6
M899		Disorder of bone, unspecified	5
S7601 (1, 2, 9) / M779 / M707 (0, 1 , 2)	#	Strain of muscle, fascia and tendon of hip (right, left, unspecified) / Enthesopathy, unspecified / Other bursitis of hip (unspecified, right, left)	5
S8310 (1, 2, 3, 4, 5, 6)		Unspecified subluxation of knee / Unspecified dislocation of knee (right, left, unspecified, right, left, unspecified)	5
S8611 (1, 2, 9) / M769 / M719	#	Strain of other muscle(s) and tendon(s) of posterior muscle group at lower leg level, (right, left unspecified) / Unspecified enthesopathy, lower limb, excluding foot / Bursopathy, unspecified	5
M722		Plantar fascial fibromatosis	4
M84359		Stress fracture, hip, unspecified	4
S29012		Strain of muscle and tendon of back wall of thorax	4
S5691 (1, 2, 9)		Strain of unspecified muscles, fascia and tendons at forearm level (right, left, unspecified)	4
M84350		Stress fracture, pelvis	3
S4300 (1, 2, 3, 4, 5, 6)		Unspecified subluxation and dislocation of shoulder joint (right, left, unspecified, right, left, unspecified)	3
S4691 (1, 2 , 3) / M67813 / M755 (0, 1, 2)	#	Strain of unspecified muscle, fascia and tendon at shoulder and upper arm level (right, left, unspecified) / Other specified disorders of tendon, right shoulder / Bursitis of shoulder (unspecified, right, left)	3
S6691 (1, 2, 9)		Strain of unspecified muscle, fascia and tendon at wrist and hand level (right, left, unspecified)	3
S699 (0, 1, 2)		Unspecified injury of wrist, hand and finger(s) (unspecified, right, left)	3

ICD-10-CM Code		Description	Total
S9360 (1, 2, 9)		Unspecified sprain of foot (right, left, unspecified)	3
S949 (0, 1, 2)		Injury of unspecified nerve at ankle and foot level (unspecified, right, left)	3
M2552 (1, 2, 9)		Pain in elbow (right, left, unspecified)	2
M2561 (1, 2, 9)		Stiffness of shoulder, not elsewhere classified (right, left, unspecified)	2
M62831		Muscle spasm of calf	2
M774		Metatarsalgia	2
M7962 (1, 2, 9)		Pain in upper arm (right, left, unspecified)	2
M7965 (1, 2, 9)		Pain in thigh (right, left, unspecified)	2
R079		Chest pain, unspecified	2
S29011		Strain of muscle and tendon of front wall of thorax	2
S299		Unspecified injury of thorax	2
S3992X		Unspecified injury of lower back	2
S4340 (1, 2, 9)		Unspecified sprain of shoulder joint (right, left, unspecified)	2
S5691 (1, 2, 9) / M779 / M719	#	Strain of unspecified muscles, fascia and tendons at forearm level (right, left, unspecified) / Enthesopathy, unspecified / Bursopathy, unspecified	2
S749 (0, 1, 2)		Injury of unspecified nerve at hip and thigh level (unspecified, right, left)	2
S8691 (1, 2, 9) / M769 / M719	#	Strain of unspecified muscle(s) and tendon(s) at lower leg level (right, left, unspecified) / Unspecified enthesopathy, lower limb, excluding foot / Bursopathy, unspecified	2
S9991 (1, 2, 9)		Unspecified injury of ankle (right, left, unspecified)	2
M542		Cervicalgia	1
M7682 (1, 2, 9)		Posterior tibial tendinitis (right, left, unspecified)	1
M799		Soft tissue disorder, unspecified	1
S161		Strain of muscle, fascia and tendon at neck level	1
S2021 (1, 2, 9)		Contusion of front wall of thorax (right, left, unspecified)	1
S298		Other specified injuries of thorax	1
S349		Injury of unspecified nerves at abdomen, lower back and pelvis level	1
S39013		Strain of muscle, fascia and tendon of pelvis	1
S449 (0, 1, 2)		Injury of unspecified nerve at shoulder and upper arm level (unspecified, right, left)	1
S499 (1, 2, 9)		Unspecified injury of right shoulder (right, left, unspecified)	1
S500 (0, 1, 2)		Contusion of right elbow (unspecified, right, left)	1
S548X (1, 2, 9)		Injury of other nerves at forearm level (right, left, unspecified)	1
S549 (0, 1, 2)		Injury of unspecified nerve at forearm level (unspecified, right, left)	1
S5991 (1, 2, 9)		Unspecified injury of forearm (right, left, unspecified)	1
S649 (0, 1, 2)		Injury of unspecified nerve at wrist and hand level (unspecified, right, left)	1
S6691 (1, 2, 9) / M779 / M720	#	Strain of unspecified muscle, fascia and tendon at wrist and hand level, (right, left, unspecified) / Enthesopathy, unspecified / Bursopathy, unspecified	1
S7310 (1, 2, 9)		Unspecified sprain of hip (right, left, unspecified)	1
S7991 (1, 2, 9)		Unspecified injury of hip (right, left, unspecified)	1
S839 (0, 1, 2)		Sprain of unspecified site of knee (unspecified, right, left)	1
S8601 (1, 2, 9) / M766 (0, 1, 2)		Strain of Achilles tendon (right, left, unspecified) / Achilles tendinitis (unspecified, right, left)	1
S8621 (1, 2, 9) / M769 / M719		Strain of muscle(s) and tendon(s) of anterior muscle group at lower leg level, (right, left, unspecified) / Unspecified enthesopathy, lower limb, excluding foot / Bursopathy, unspecified	1
S900 (0, 1, 2)	#	Contusion of ankle (unspecified, right, left)	1
T148	#	Other injury of unspecified body region	1

# Indicates where multiple taxonomy codes were used due to the pre-set ‘injury type’ options within the standardised reporting form not directly aligning with one ICD-10-CM code, therefore the incident injury could be considered as any one of the code options identified (e.g., tendon rupture / tendinosis / bursitis).
