# Supplementary material for: A Comprehensive Analysis of Injuries During Army Basic Military Training
Source: Mil Med. 2022 Jul 4;189(3-4):652–60. doi: 10.1093/milmed/usac184 (PMC10898870; doi:10.1093/milmed/usac184)
Supplement: usac184_Supp [file usac184_supp.zip › Supplementary Table 2.pdf]

Session Type	Total	Overuse	Trauma	Unspecified
Endurance	78	70	4	4
Strength	55	43	10	2
PESA + Occupational Tasks	41	24	16	1
RDJ + Obstacle course	33	17	16	0
PFA + BFA	15	10	5	0
Swimming	14	10	4	0
Nutrition / lift + carry	11	6	5	0
DSTG Testing	5	4	1	0
Introduction PT	0	0	0	0
Recovery	0	0	0	0
Total	252	184	61	7

†PESA = Physical employment standard assessment; RDJ = run, dodge, jump; PFA = Physical fitness assessment; BFA = Basic fitness assessment; DSTG = Defence Science and Technology Group; PT = Physical training
